# News and Notes

**Published:** 1899-05

**Authors:** 


### *NEWS AND NOTES.*

ATLANTA's municipality has passed a "spitting ordinance," prohibiting expectorating on the sidewalks and in all public places.

DR. T. E. COLLIER, one of the oldest physicians in Atlanta, died in this city on April 13th. He had been in feeble health for some time, and his death was not unexpected.

THE Atlanta Dental College graduated a class of forty-five men on the night of March 30th, and on the night of April 3d the Dental Department of the Atlanta College of Physicians and Surgeons graduated a class of thirty-two.

DR. DAN H. HOWELL died in Atlanta on April 16th. Dr. Howell was for a number of years editor and owner of *The Southern Medical Record*, and was an active member of the Georgia Medical Association, of which body he was once the Secretary. For the last few years Dr. Howell had been in ill health, so that his death was not unexpected.

DR. A. R. ROYALL, one of the leading physicians of Abbeville, Ga., died in that city on March 24th.

DR. C. S. NEWTON, of Madison, Ga., but formerly of Atlanta, died in the former city on April 19th. He was at the supper-table when the stroke of death came.

DR. F. H. BLOODWORTH, one of the young physicians of the State, and at the time of his death one of the city physicians in Savannah, Ga., died in that city on March 27th.

DR. J. A. STEWART, one of the oldest and one of the most prominent physicians in the State, died at his home in Conyers, Ga., on March 28th. Dr. Stewart had served his country in the State legislature, and was an active Confederate veteran. He was a man of prominence.

---

THE daily press informs us that Dr. Bra, of Paris, has found and isolated the germ of cancer. We wait for further authentic information.

---

DR. A. MARTIN, a celebrated gynecologist of Berlin, removed to Greifswald on April 1st to assume the chair of gynecology in that University.

---

DR. HENRY P. NEWMAN of Chicago, has been elected to the Chair of Gynecology recently made vacant by Dr. Etheridge in the Chicago Polyclinic.

---

THE Richmond, Va., medical journals, of which there are four, must certainly be on very friendly terms, judging by the manner in which they all print the same original articles.

---

THE Birmingham Medical College of Birmingham, Ala., held its commencement on the evening of March 29th. The number of graduates was six; among which number was one woman graduate.

---

IT is reported that Dr. Schenck, of Vienna, recently made famous by his alleged method of determining the sex of the fetus, has been reprimanded by the Vienna Medical Society for publishing his investigations and conclusions first in the secular press instead of in a medical journal.

---

DR. ANGELO KNORR, a *Privat-docent* in the veterinary school of Munich, died in that city on February 22d. His death was due to acute glanders contracted in the course of an experimental research on that malady. Germany and Austria have both been unfortunate in this direction.

ONE of the Spanish Barracks in Havana has been completely renovated, cleaned, remodeled and fitted out as a yellow fever hospital. Major Ducker, brigade surgeon, U. S. V., has applied to Surgeon-General Sternberg for immune nurses, stating that their quarters are ready for occupation.

WE are sorry to see that the *Journal of the American Medical Association* is taking advertisements of the Guarantee Association order. Companies from Chicago which insure you $125 a month for life income are to be watched with care, for there is more than one tricky thing in the Windy City.

GOVERNOR ROOSEVELT has signed the bill passed by the legislature prohibiting bicycle racing for a greater period than twelve hours in twenty-four hours, so that in future the public will be spared the melancholy spectacles of exhaustion that have hitherto characterized the six days' races at Madison Square Garden.

THE *British Medical Journal* informs us that the Ophthalmological Society of the United Kingdom has resolved to admit women to its membership. This Journal adds, "women have now the same legal status in the medical profession as men, and it is just and wise that the right hand of fellowship should be held out to them."

THE biggest medical contract on record is that given by General Guy V. Henry, governor of Puerto Rico, to Dr. Azel Ames of Massachusetts. The doctor has been ordered by the general to vaccinate every one of the island's 1,000,000 inhabitants. It will take him and his assistants six months to complete the contract and the cost will exceed $100,000.

NEWS comes of an outbreak of the plague at Jeddah, the port of Mecca, on the Red Sea. Attempts to enforce quarantine regulations at Jeddah met with great opposition from the pilgrims, and it is said that the troops sided with them. This outbreak of the plague will probably be followed by the establishment of the disease in Egypt, Syria and Turkey.

---

NASHVILLE must be having an exodus of physicians, judging by the *Journal of the American Medical Association.* In its issue of April 8th, under "Change of address," nine physicians are given as moving away from that city. It may be that they were newly graduated medical students, yet others who read the list had no way of judging whether such was the case. Nashville seems to be in luck.

---

PROF. JOHN URI LLOYD, of Cincinnati, Ohio, has examined the new remedy, "Husa," the so-called opium habit cure recently advertised extensively by W. W. Winthrop, A.M., M.D., of Fort Worth, Tex., who claims to have discovered the wonderful medicinal properties of this plant in the wilds of Florida. Prof. Lloyd finds that the solutions of "Husa" sold by Dr. Winthrop are nothing more than various strength solutions of morphine and alcohol, and that the wonderful herb business is a "fake."

---

THERE is a great difference between the United States and Russia in the number of graduates in medicine out of the total number of medical students. According to the *British Medical Journal,* during 1898 the degree of Doctor of Medicine was conferred by the University of Moscow on fourteen, by the University of Charkoff on five, and by the University of Kieff on three candidates. The number of students of medicine in the three universities severally was on January 1st, 1899, as follows: Moscow, 1,294; Kieff, 895; Charkoff, 709.

PROFESSOR KOCH is said to be fitting up an expedition to go to South Africa, especially to German East Africa. His intention is to study malaria and kindred diseases more fully, especially with an eye to prophylaxis, immunization, and treatment. With malaria under control Professor Koch insists that the tropics would be perfectly habitable for Europeans and Caucasians generally. He aims also to investigate more fully and locate the exact limits of the plague focus that he found on the shores of Lake Victoria Nyanza during his previous visit to Africa.

---

THE Physicians' Municipal League, organized at Cleveland, Ohio, has been eminently successful in attaining the objects which prompted organization. A meeting was held March 23d, at which addresses were delivered by four speakers interested in the movement to divorce municipal affairs from politics. The meeting was enthusiastic, over 200 members of the best of the Cleveland profession being in attendance. All classes of citizens have agreed that the action of the league delivered the hardest blow to machine politics that has ever been witnessed in this city, because on the face of it the movement was entirely disinterested and not to be discounted by the machine politician. The league was the means of arousing the apathy of the good citizens, so that following its action the newspapers began to take a decided stand and public mass-meetings of citizens were held. It having been the means of arousing the public conscience, success in throwing off the shackles of boss rule from the city was assured. At the election on April 3d, the self-sacrifice of the physicians was amply rewarded. While the nominal republican candidate for mayor, against whom the fight was made, was defeated by nearly 3,000 votes, the rest of that party's ticket was elected by majorities ranging up to 9,361. A more direct and stinging rebuke to "machine" methods, or a more gratifying triumph of the good elements of society, could not be asked. Further, on all sides was heard the comment that the action of the medical profession was what carried conviction of municipal corruption home to the mass of voters.

